# Sardinian deer increase feeding diversity within sheltering vegetation in a fragmented Mediterranean landscape

**DOI:** 10.1038/s41598-024-80818-z

**Published:** 2024-12-18

**Authors:** Sabine Aboling, Fahad Ahmed, Gunnar Kreisel, Josef Kamphues, Maria Grazia Cappai

**Affiliations:** 1 Institute of Animal Nutrition, University of Veterinary Medicine, Hannover Foundation, Bischofsholer Damm 15, 30163 Hannover, Germany; 2 Nutrition Innovation Centre for Food and Health (NICHE), School of Biomedical Sciences, Ulster University, Coleraine, BT52 1SA UK; 3 Institute of Philosophy, Leibniz University, Im Moore 21, 30167 Hannover, Germany; 4 Chair of Animal Nutrition, Department of Veterinary Medicine, University of Sassari, Via Vienna 2, Sassari, 07100 Italy

**Keywords:** Crop damage, Deer, Evenness, Foraging, Sardinia, Shelter, Trampling, Zoology, Ecology

## Abstract

This study was carried out to investigate the feeding behavior of Sardinian deer roaming within a site of community interest (SCI, ITB042250), on Sardinian Island (39° 51’ N 8° 45’ E). Crop fields bordering the natural environment (wood forest and Mediterranean macchia, where the reserve of Sardinian deer partly overlaps) were monitored and turned into a living lab. The interest on wildlife-related crop damage poses a significant economic challenge, while anthropogenic pressures, such as urbanization and agricultural practices, increasingly impact wildlife by limiting habitat, feeding source diversity and access, and space distribution. In view of this, dietary habits of a group of 25 Sardinian deer (*Cervus elaphus corsicanus* Erxleben, 1777) were studied over an area of 45 hectares, in spring and autumn over two consecutive years. Within six representative sites (habitats) of investigation (SOI = 3 ha), two transects of 20 × 10 m per SOI, amounting to 240 m², served as parts of these sites, respectively, and were analyzed as to floristic composition and dominating plants. The number of freshly browsed/grazed plants by missing shoots was counted and assumed as signs of foraging activity of the Sardinian deer. The evenness of the dietary preference per habitat was calculated with the number of missing shoots and the number of plant species selected. The Mediterranean macchia accounted for the highest species (*n* = 76); crop field the fewest species (*n* = 19). Plants showing foraging signs by Sardinian deer ranged between n. 5 out of 46 species (10.9%) in the fallow and n. 6 out of 19 species (31.6%) in the crop field. Complementary feeding based on the availability of floristic diversity revealed to align to the typical foraging behavior of intermediate feeder in all SOIs: the higher the species, the higher the number of foraged species (ρ = 0.86). Sardinian deer foraged on n. 35 out of 129 plant species present (27.1%) within the whole transect area. Data highlight two feeding behavior: 1)high evenness-feeding (less foraging on high number of plant species, respectively: fallow, crop field, macchia border); 2) low evenness-feeding (large foraging on few species, *Avena* or *Cytisus*: field, bordering both macchia and fallow, macchia).The Sardinian deer fed on every SOI, with highest consumption in the macchia and least in oat open field. The feeding behavior of Sardinian deer confirms the ability to adapt to the varying vegetation in each habitat, respectively, by seeking energy and nutrients on the one hand and shelter on the other, by limiting the preference for crop field center.

**Supplementary Information:**

The online version contains supplementary material available at 10.1038/s41598-024-80818-z.

## Introduction

The spatial distribution of herbivoresand frugivores varies worldwide, manifesting in discrete social systems, physiology and feeding habits co-evolved with the environment^1–6^. The deer (*Cervus elaphus* L.,1758) is a cervid, with wide Palearctic distribution, inhabiting large parts of the European continent, as well as Afro-asiatic areas. The wide distribution of deer points to the extraordinary adaptation to diverse habitats^1^.Deer contribute to a wide array of ecological functions, including nutrient cycling, control of vegetation, and biodiversity conservation, as feed dispersal^7–9^. In view of this, deer natural diet and feeding preferences are crucialin maintaining theirexistence^2,3^. Under natural conditions, the diet of the deer is strongly influenced by seasonal and geopedological characteristics of the territory and available feeding sources, as major determinants to plant growth and production^10^. Accordingly, certain large herbivores reciprocate to heterogeneity by being both generalist feeder, or grass and roughage eater (grazers), as well as being selective towards a variety of specific parts of plants (scrubland and woodland) as concentrate selectors.The combination of both foraging strategies earns the deer to be classified as “intermediate- or mixed-feeder”^11,12^. Such foraging habit is characterized by year-round feeding upon all kinds of available vegetation such as buds and fruits and even lichens, altogether described as energy dense feeds or concentrates^12^ (in contrast to fibrous material, less energy dense). Deer can move from forests to croplands, showing wide space occupancy and distribution, also thanks to diet diversity. Mixed feeding along with foraging^13^ extent, should be monitored for forest benefits and population size or, as in the case of a fragmented landscape, as well as to assess crop field damage^14,15^ especially if issues about sustainable rural farming is challenged.The election diet in the nature follows habitat puculiarities in terms of species and subspecies^16^ accounting for nutritional quality and anti-nutritional properties of plants^16–18^. Furthermore, individual energy and nutrient requirements are related to physiologic state, gender, social and reproductive dynamics, thus displaying an expected variability^3,19,20^. Foraging patterns and overgrazing may increase the biomass volume of less desirable or inedible plants^18,21,22^.It was hypothesized that the assessment of florisitic diversity and composition within the SIC area, where the red deer of Sardinia were recently relocated, would contribute to the advancement of knowledge for monitoring the foraging activity based on feed preference. Understanding deer foraging behavior and its determinants is essential, as it would help in developing accurate models for estimating the use of feeding sources^23,24^. In view of this, the potential feeding competition between wildlife and farmed animals may induce economic pressure for crop production and food producing animals^25^, as well as for agroeconomics and sustainability of farmland, especially if the progressive shrink of natural surface available to wildlife is also considered, following climate change and disasters (like fire prone areas).

So far, extensive knowledge regarding ungulate feedinghabits has been gathered forroe deer (*Capreolus capreolus* L.)^25,27^and red deer from central Europe, Great Britain and Iberic peninsula^28–31^. However, to date, information about the feeding ecology of Sardinian deer (*Cervus elaphus corsicanus*Erxleben, 1777), appears to be limited to a few papers^1,31–34^. Sardinian deeris a recognized subspecies of *Cervus elaphus* L. and was originally described as an endemic species (IUCN, 1990), while no longer being included within the IUCN list^35^,currently of least concern and completely protected by the Habitat Directive^36^. The Sardinian deer was once uniformly distributed all over Sardinia island^38^.However, its actual distribution is greatly restricted to South-West mountainous regions, and mountains on the South-East and West coast of the island^1^.“One Deer, Two Islands” LIFE project in 2013, allowed Sardinian deer to shift from 4.270 individuals in 2014 to 10.500 individuals in 2019^39^.The geographical distribution of Sardinian deer will presumably increase on the island, for large predators and venomous animals are absent on the Sardinian Island and deer hunting forbidden. In this scenario, the rationale behind the investigation acknowledges the need to define the role of Sardinian deer as foragers in fragmented landscape of the SIC area, for which feeding habits urge to be detailed. This investigation was carried to (a) catalogue and quantify the diversity of plant species that red deer forage on, and (b) explore the feeding behavior with regard to vegetation structure and floristic diversity of a fragmented landscape in one of major Mediterranean islands where the deer subspecies live.

## Materials and methods

### Study site

**Fig. 1:**
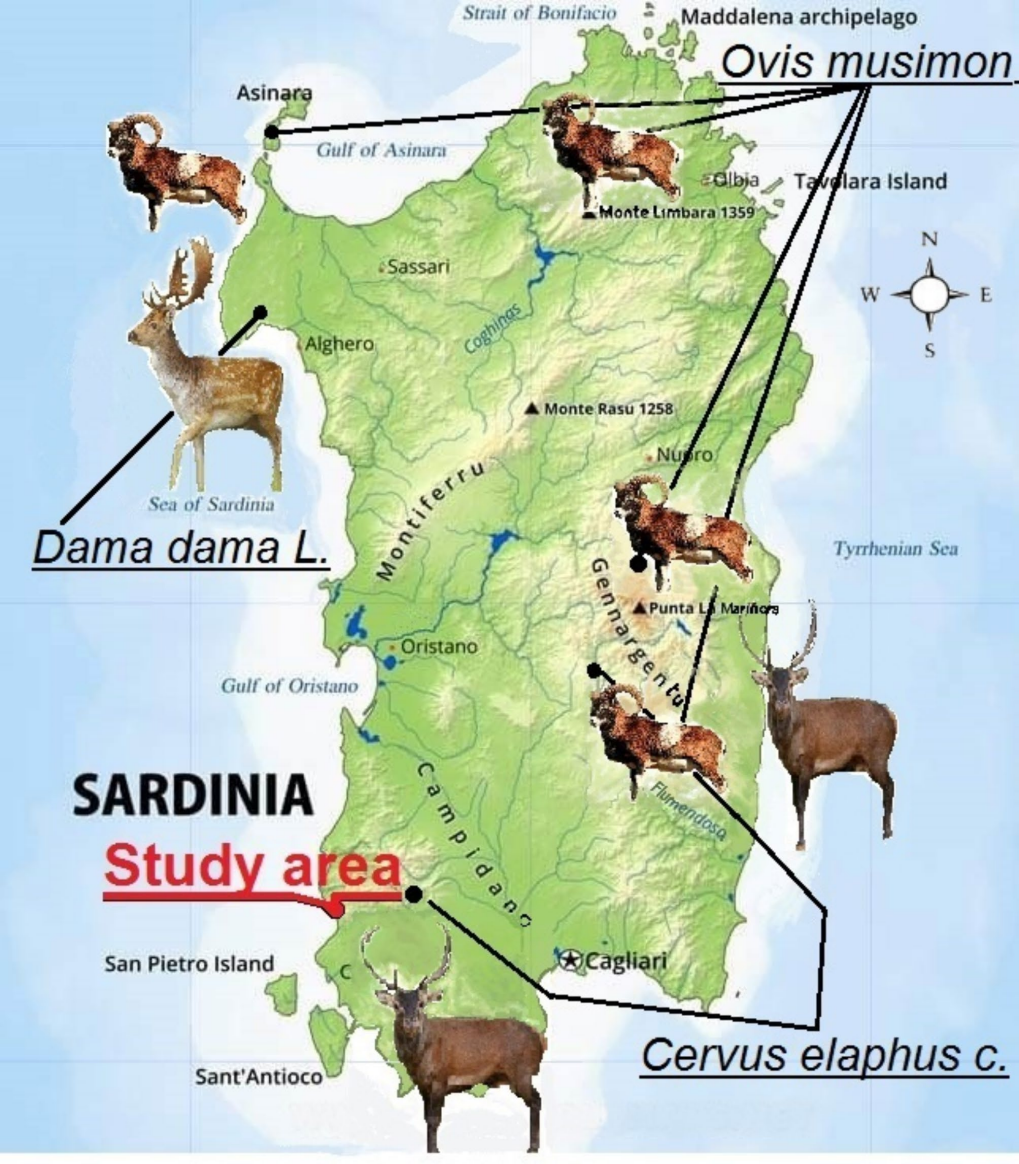
Map of Sardinia. Species of large wild ruminants and distribution over the island. The figure was created with Paint software Microsoft Windows10 ^®^.

The study was carried out on the Island of Sardinia (Italy) which is one of the major island of western Mediterranean Sea (38° 51′ N 41° 15′ N and 8° 8′ E and 9° 50′ E). Field observations were carried out within a site of community interest (SCI, ITB042250). In particular, the group of deer in the focus of this study move in the area from “*Brachypodietalia* dune grasslands with annuals (habitat code 2240; ha 5.32) to “*Quercus ilex* and Quercus rotundifolia forests” (habitat code 9340; ha 2.42), across “ Coastal dunes with *Juniperus spp*.” (habitat code 2250; ha 12.16),“Wooded dunes with *Pinus pinea* and/or *Pinus pinaster*”(habitat code 2270; ha 67.74). Sardinia covers an area of 24.000 km^2^ with a long 1.900 km coastline. The landscape of Sardinia consists of geomorphological variability with mountains and hills as largest portion (65%) followed by plane areas with a variety of vegetal *taxa*, some of which are endemic and unique worldwide. The typical Mediterranean climate is characterized by dry and hot summer and moderately wet and rainy winter. The average annual precipitation ranges from 411 to 1215 mm with mean annual temperature ranging from minimum 11.6°C to maximum 18.0°C. The land in plane areas is utilized for agricultural purposes (cropland and extensive farming, chiefly small ruminants and to a minor extent by beef cattle, along with few areas destined to intensive farming systems for dairy cattle) which represents approximately 50% of the land use. Predominantly covered by shrubs (typical Mediterranean macchia), mountain forestlands include *Fagaceae* (namely *Quercus pubescens*, *Quercus suber* and *Quercus ilex*) surrounded by pastures^40,41^. Other wild herbivores live on Sardinian Island, namely the Mouflon (*Ovis orientalis musimon*, Pallas 1762) and the Fallow deer (*Dama dama* L., 1758, allochthon as recently introduced), mainly distributed in different areas of the island (Fig. 1). Other non-ruminant wild and feral animals of megafauna are represented by Giara horses^42^ and Asinara donkeys^43^, which however do not occupy At this regards, it is also to point out that no large predators exist on Sardinia island, where the largest mammalian wild carnivore is the Sardinian fox (*Vulpes vulpes ichnusae* Miller, 1907^44^), followed by the Sardinian wildcat (*Felis silvestris lybica* Forsters, 1780^44^) and the Sardinian pine marten (*Martes martes latinorum* Barret-Hamilton, 1904^44^). In addition, no venomous snakes, insects or arachnids represent a life-threat for humans and animals in the wild on the whole island.

### Data recording

**Fig. 2:**
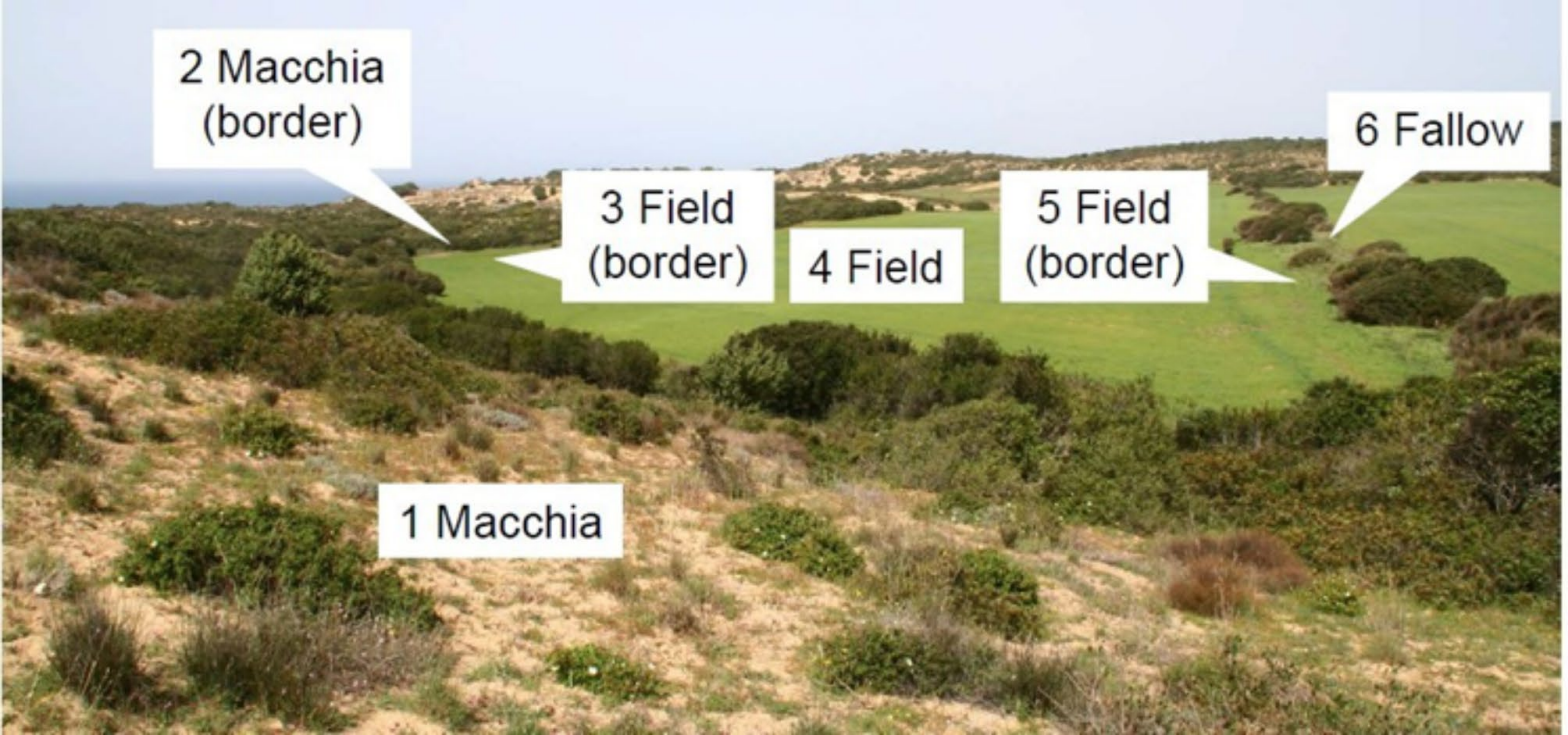
Study area with SOI (1, 2, 3, 4, 5, 6) examined.

Field work took place in spring and repeated observations carried out autumn. Representative habitats of about three hectares and adjacent to another habitat (Fig. 2) were chosen. In each habitat, two transects of 20 × 1 m (simple repeat) were established and surveyed with GPS, amounting in total to 240 m² of observation and systematic identification of plant species. Counting of missing shoots to document foraging activity by means of transects was carried out by positioning 10 squares a 2 m².Botanical data accounted forthe estimation of vegetation coverage (%) by oat (% *Avena sativa*).Moreover, dominating as well as total plant species were systematically classified. Browsed vegetation (bushes) or grazed (herbaceous plants) by deer were monitored by careful inspection of clear signs of bites and missing parts, such as leaves, twigs, or shoots, which indicated foraging activity. Fresh signs of browsing, such as recently nibbled or broken shots, were identified through direct visual inspection. The number of browsed shoots was then systematically counted for each plant species within the square transects, providing a semi-quantifiable measure for estimating feeding activity.

### Evaluation on site

For analytical purposes, the study area was spatially categorized to identify different feeding sources composition according to land use, as shown in Fig. 2. The site of investigation SOI-1 corresponded to Mediterranean macchia, exclusively as “distant” from the seminative; the SOI-2 accounted macchia bordering seminative field including Mediterranean shrubs; SOI-3corresponded to borders and field; SOI-4was field center and SOI-5was field close to border. The fallow is SOI-6, and it represented arable areas without sowing. In each SOI two transects sided 20 min length were chosen randomly, further divided into 10 squares of 2 m² surface.

### Analysis of data

Data per habitat comprised six records: two transects per SOI, with two observation periods (spring and autumn).

To determine to which extent deer expressed the feeding preference among available plant species, the evenness index was used, as an ecological quantitative index representative of the uniformity of proportion of plant species preference within the vegetation^45^. The higher the number the more the contributing species. A low index indicates that one or few plants are dominating over others. Evenness index (Ei) was calculated by the number of foraged shoots and the number (variety) of foraged species. Thus, it closely documents whether the deer relied on all plant species equally (high Ei) or preferred one or few plant species (low Ei).The Ei value ranges from Ei = 0 to Ei = 100,as broadening from none to highest diversity, respectively.

- Indicator for feeding diversity from 0 to 100.
- Browsing intensity of all species even (equally balanced) = 100.
- Browsing intensity constructed on one species = 100.

$$- \sum _{{i = 1}}^{n} p_{i} lnp_{i} \div ln_{n} \times 100$$

n, Number of browsed shoots per transect.

p_i_, Relative portion of browsed shoots per species and transect [%].

(100%= the total of the browsed shoots per transect)

Pearson’s correlation test was carried out to assess whether the choice of plant species could be proportional to plant species diversity (ρ < 0.300 = weak correlation; 0.300 < ρ < 0.600 = mild correlation; 0.600 < ρ < 1.000 strong correlation; +ρ or –ρ, positively or negatively correlated, respectively).

## Results

**Fig. 3:**
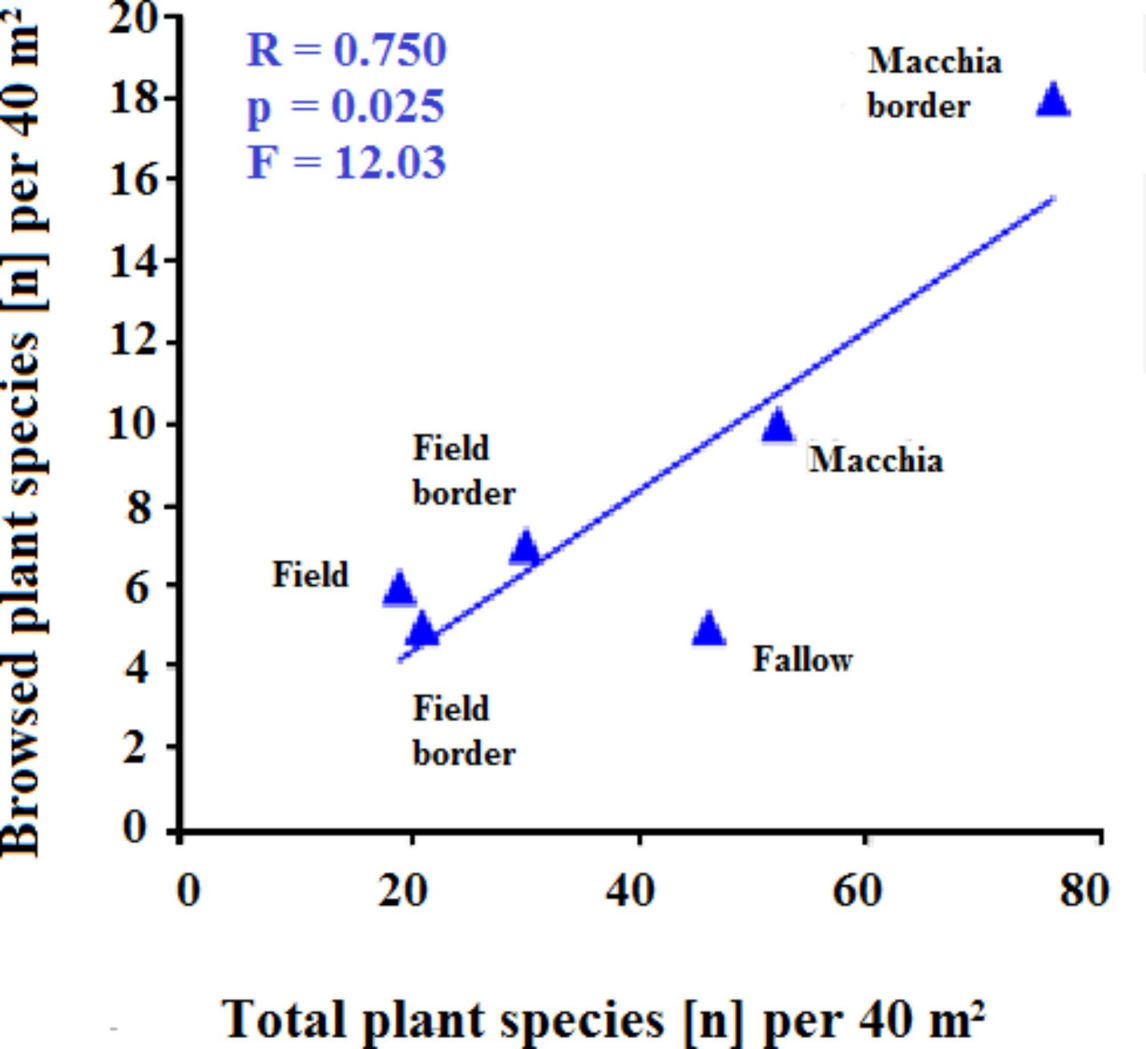
Correlation between number of available plant species and visited/ingested.

The richest habitat in plant species turned out to be the Mediterranean distant macchia (SOI-1), followed by Mediterranean macchia bordering the seminative (SOI-2), with 76 plant species (Table 1). In contrast, the seminative center (SOI-4)contained only 19 species. In total, 35 plant species out of 129 identified (27.1%) showed signs of deer foraging within the selected 240 m² of the total transected areas. Although the deer ate a minority of the species present there was a positive correlation between the two parameters (Fig. 3).

**Table 1:** Parameters of floristic and grazing diversity in the habitats. Eating fewer shoots of more species (italic) and eating of more shoots of lesser species (bold).

Anthropogenic influence		Natural	Natural, artificially influenced	Artificial, naturally influenced	Artificial	Artificial, naturally influenced	Natural, artificially influenced
No of habitat		1	2	3	4	5	6
Habitat		Distant Macchia	Macchia bordering field	Field bordering Macchia	Field	Field, bordering fallow	Fallow
Total species (n)		52	76	21	19	30	46
Ingested species (n)		10	18	5	6	7	5
Eveness		**21.9**	*71.6*	**4.4**	*64.6*	**19.4**	*79.4*
Ingested shoots, sum (n/40 m²)		5261	382	1041	219	1805	20
Ingested shoots per species (n/40 m²)	*Cytisus salzmannii*	4620	.	.	.	.	.
	*Polycarpon spec.*	.	90	.	.	.	.
	*Rumex bucocephalus*	.	139	.	.	.	.
	*Avena sativa*	.	.	1030	129	1635	10
	*Spergula arvensis*	.	.	.	51	.	.
	*Vicia villosa*	.	.	.	.	.	3

In the detail for the distinct habitats, Sardinian deer left traces of eating on a few between 5 and 18 plant species (mean = 8.5) out of the total species, ranging between 19 and 76 (mean = 40.7) per habitat. Deer ingested 5 out of 46 species in the minimum in the fallow (SOI-6; 10.9%) and 18 of 76 species in the maximum in the Macchia bordering seminative (SOI-2; 23.7%). The range of plant species preferred as feeding sources was between5 out of 46 species (10.9%) on the fallow (SOI-6) and 6 out of 19 (31.6%) on field (SOI-4).Typical Mediterranean macchia in SOI-1 was dominated by shrubs such as *Cytisus* and *Ephedra* with a mean vegetation coverage of 40%, varying between 10 and 90%. Between the woody plants, herbaceous *taxa*, such as *Hieracium*,* Lithospermum*,* Silene*, and *Misopatesorontium*, were identified with plants as high as between 2.5 and 3 mt.

The second unit was macchia bordering seminative (SOI-2) with *Rhamnus latifolius* as woody species in common with the typical macchia. This type of macchia accounted for more herbaceousspecies leading to an overall higher vegetation coverage of 70%. More in detail, plants such *Bromus sterilis*,* Hieracium*,* Lupinus angustifolius*,* Polycarpon*,* Rumex bucocephalus*,* Senecio*,* Silene*, and *Sonchus arvensis*were identified.

As against the shrub-dominated macchia, the third unit comprised open sites and wascomposed of different kinds of field habitats (SOI-3,4,5)on the one hand and fallows on the other hand (SOI-6). The sites shared numerous herbaceous species such as *Avena sativa* as sown crops as well as *Sinapis arvensis* and *Anthemis arvensis* as weed. Moreover, *taxa* such as *Asparagus*,* Chrysanthemum segetum*, *Raphanus*, and *Vicia villosa*, likewise feed plants, were typical here. Mean coverage of the whole herbaceous vegetation inclusive non-feed plants, was between 25 and 55% on the fields or their margins, and 90% on the fallow.

Deer appeared to rely on every type of habitat (natural vs. crop and borders) for seeking feed.

**Fig. 4:**
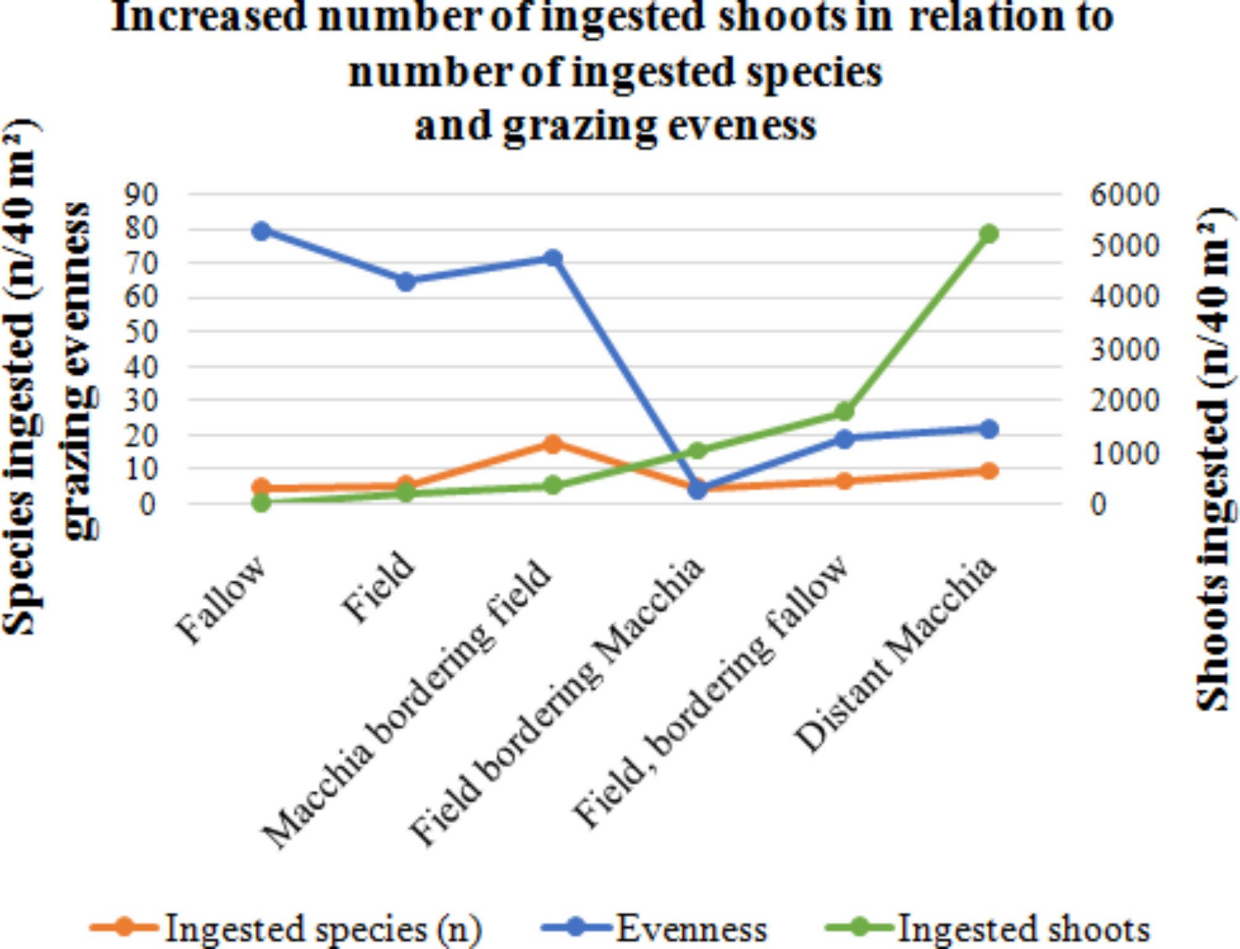
Parameters of diversity in grazing ecology: number of species ingested, grazing evenness and number of ingested shoots.

Overall, two kinds of foraging behavior could be derived from data: feeding less than 400.

shoots of several plant species and feeding in total more than 1000 shoots of one or few plants.

species. The first kind of grazing behavior (eating less of numerous species, high evenness; “high evenness-grazing”) was seen in three habitats (fallow, field, macchia bordering field; Fig. 4).

For example, on the fallow (SOI-6), the deer preferred a low number of shoots (*n* = 20) and ate from each species that has been elected as feed plant shoots that numbers varied not very much (high evenness index = 79.4). In this example (SOI-6) deer preferred altogether 20 shoots that were partly from *Avena sativa* (10 shoots), partly from *Vicia* spp. (3 shoots) and from three further plant species, respectively (remaining 7 shoots).

In contrast, the second kind of grazing behavior (eating much of few species, low evenness; low.

evenness-grazing”) was observed in the other three habitats (field bordering macchia, field, bordering fallow, distant macchia). On the macchia (SOI-1), the deer preferred the highest number of shoots (*n* = 5261) and selected mainly *Cytisus salzamanii* (*n* = 4620) of altogether ten feed plants.

The small rest of preferred shoots (*n* = 641;14%) belonged to the whole rest of nine further feed plant species (low evenness index = 21.9). Only two plant species chiefly involved in low evenness-grazing: *Avena sativa* on the fields (SOI-3 and 5) and *Cytisus salzamannii* in the macchia (SOI-1).

The choice strongly depended upon the floristic diversity of the habitat since the more species a habitat possessed, the more the deer visited larger number of species (*r* = 0.86).

## Discussion

The interaction between wildlife and the environment in natural habitats is essential for ecological balance, when perturbation is minimized. The main outcomes of this field study were supported by the ideal conditions to assess the impact on the feeding habits of the Sardinian red deer, within a fragmented landscape of a SIC area, where the interference from human and livestock is reduced to a minimum. As for the reduction of such biases, and the special permission obtained to carry out the experimental activities, this field study represented the ideal condition for the so called “open living lab”. Key findings point to Sardinian red deer capable of adapting the selection of feeding sources in the different habitats (SOIs 1 to 6) by either (1) adopting low evenness-feeding while they clearly exploit the floristic diversity, or by (2) high evenness-feeding as well as (3) complementary usage of floristic diversity. All strategies are spatially combined and applied on distinct habitats, respectively, and reveal that Sardinian deer is adapted to feed in the fragmented environment. Such feeding plasticity earns the deer the potential to spread, thanks to the possibility offered by different types of vegetal composition. Indeed, grass and roughage eaters or grazers represent about the 25% of nearly 150 ruminant species known^46^. The intermediate feeders are herbivores choosing the mixed diet and capable of the highest adaptation to available feeding sources in the environment^46^. As well, balancing the diet is important, in relation to biodiversity of vegetation^46,47^. In detail, large intake of shoots along with the preference of a certain plant species as part of (1) low evenness-feeding occurs in the field margins (via grazing) and macchia (via browsing), however, not in the open oat fields. Large feed intake in these habitats probably largely goes back to safety issues. The innate behaviour of the deer at eating accounts both for nutritional attractive natural sources and shelter to protect from potential predators. The act of eating represents a weak point for a prey in nature, because when the herbivore is eating, that is when it is more vulnerable to threats (predators). The shape of the environment makes the difference, especially vegetation height for some herbivores, as well as access to natural freshwater sources (rivers or ponds). Sardinian red deer showed to prefer quantitatively less (< 1000 shoots) when grazing in open fields, fallows and field-adjacent macchia. Comparatively, our findings agree with those on European red deer that choose 29% (145 plant species) out of the whole flora^48^. This is comparable to roe deer (*Capreolus capreolus*) and European hare (*Lepus europaeus* L.) that select in general only 35% of all plant species present in one habitat^49^.

Considering the geographic isolation of Sardinia and lack of large predators like bears and wolves, the rise in population of red deer may lead to require future management^50^.The knowledge about the feeding behavior contributes to reconsider the role of the roaming deer in determining crop damage, for cultivated fields do not represent the first feeding choice, as highlighted by results of our trial. Thus, spatial distribution and competition for feedstuffs would also suggest to consider that access to natural feeding sources may rather be shared by domestic goats as browsers, than sheep and cattle, usually foraging in open pasture. However, in view of the risk of diffusive diseases^51,52^ that deer share with domestic ruminants, additional concerns of sanitary and economic value^51,52^may rise. Landscape planning appears strategic for the rational preservation of natural habitat, along with conservation management of wildlife and biodiversity of vegetation. Rational use of the territory would therefore prevent the overlapping of browsing areas^53^, once foraging habits are known and the knowledge of feeding preferences of deer may help in adequately selecting the most appropriate translocation areas, within management programs. Reduction of conflicts for spatial competition, are in fact particularly important on islands like Sardinia, for biodiversity conservation on one side and to warrant sustainable farming on the other. Thus, the understanding of dispersion and home range is essential since they are major elements in ecosystems^54,55^. The behavior of cervids following release is regulated by landscape spatial patterns^56^ and by disturbances caused by humans^57^. The migration and dispersion of reintroduced animals can be characterized by larger region of occupancy, or a small area with strong release-site fidelity^54,57^. In view of our results, the expression of the feeding behavior of Sardinian red deer would serve as a basis for future management programs which may take into account the peculiarities of Sardinia island, accounting for adaptation to floristic diversity and landscape composition. Agriculture practices may consider the “redirection” of roaming deer groups, by appropriately managing crop field borders as strategies to limit potential trampling and minimize the contact with domestic animals.

## Conclusion

Deer demonstrated to adopt the mixed feeding behavior in a fragmented landscape, being capable of balancing the preference for dominating plants according to energy density in different habitats. When natural and cultivated habitats were present, the deer showed to narrow the feeding preference to a 27.1% of the total plant species available and identified on the whole study area. Foraging diversification (high evenness vs. low evenness), depending on floristic diversity of the habitat, is also the expression of the selection for sheltering vegetation, used by deer as innate behavior for escaping from predators, meanwhile browsing. Based on findings, it is reported that the feeding range and foraging type of deer is low on crop fields, which are not as attractive as expected, unless in the case of bordering seminative close to sheltering natural vegetation, like the Mediterranean macchia, in our case. The fragmentation of natural habitat requires to be managed, and adequate knowledge of landscape composition and foraging behavior pivotal, to identify the most suitable areas into which deer can be translocated. Of importance, the preservation of natural corridors for Sardinian red deer appear crucial, to allow spatial distribution in the natural environment, by considering the innate behavior while roaming. In view of the shape and composition of natural vegetation, agricultural practices may point to redirect animals from the center of crop fields to border with appropriate rural planning and sowing methods. In conclusion, the detailed knowledge of Sardinian deer foraging and floristic diversity of the natural environment should be considered when conservation plans are set up, to manage the risk of spatial conflict between cropland and natural habitat.

## Electronic supplementary material

Below is the link to the electronic supplementary material.

Supplementary Material 1

## Data Availability

Data is provided within the manuscript or supplementary information files.
